# The Learning Curve in Digital Replant Surgery: 46 Prospectively Collected Cases From a Single Surgeon Over a 10-Year Period

**DOI:** 10.7759/cureus.66133

**Published:** 2024-08-04

**Authors:** Joshua W Hustedt, N Ake Nystrom, Lloyd Champagne

**Affiliations:** 1 Orthopaedic Surgery, University of Arizona College of Medicine, Phoenix, USA; 2 Plastic Surgery, Stavanger University Hospital, Stavanger, NOR; 3 Plastic Surgery, Arizona Center for Hand Surgery, Phoenix, USA

**Keywords:** limb replantation, replantation, plastic surgery, hand surgery, microsurgery, digital replantation

## Abstract

Purpose: Digital replantation is a technically difficult microsurgery requiring significant surgical skill. The aim of this study was to investigate postoperative outcomes associated with the surgical learning curve for microvascular digital replantation.

Methods: A prospectively maintained surgical database of consecutive patients who underwent digital replantation from 2002 to 2012 was reviewed. All cases were performed by a single surgeon and began immediately after the surgeon’s fellowship. A total of 46 patients were identified. Outcomes of digital replantation were tested for association with time since fellowship, total microvascular operative experience, and location and type of injury.

Results: Overall, 38/46 (82.6%) of patients underwent a successful digital replantation. There was a significant difference between survival percentages over the years (p=0.04), with improvement seen over time. Total microvascular experience was significantly associated with successful outcomes (p<0.001). After 100 hours of microvascular experience, there was a significant increase in the survival odds ratio (OR 8.5, 95% CI 1.5-47.9). Crush and thumb injuries were more likely to have detrimental outcomes.

Conclusions: There was marked improvement in replant survival over time, with a significant increase in odds of survival after 100 hours of microvascular experience. One hundred operating hours under the microscope occurred around 2 years in practice for this high-volume surgeon. There is strong evidence that a steep learning curve occurs in microvascular digit replantation surgery.

## Introduction

Since the first described successful thumb replant in 1965 digit replantation has grown to yield high success rates in the developed world [1-3]. Recent meta-analyses of digit replants report success rates in the 80-90% range, depending on indications [4,5]. Advances in microvascular surgery have expanded the role and indications for digital replantation, making replantation a viable option in the setting of a severed digit [6].

Despite the growing interest and success of digit replants in the developed world there has been a slow decline in the rate and success of digit replants in the United States [7-10]. Recent meta-analyses and retrospective reviews have pointed out that a majority of microvascular surgical progress in digital replantation is being undertaken in Asia [5,11]. In fact, a review of the literature yielded only five prospectively collected studies on digital replants in the United States in the last 20 years [11-15].

Furthermore, fewer and fewer US hand surgeons report experience with digit replantation. A recent survey of the American Society for Surgery of the Hand (ASSH) members reported only 56% of surgeons perform replants [16]. Of those who do perform replants, the majority performed fewer than five replantations per year. While the reasons for this decline are likely multifactorial, reports have suggested a declining number of amputations, declining reimbursement for higher complexity cases, decentralization of replants away from high-volume centers and providers, and increased selectivity for attempted replantation as plausible causes for the decreased performance of digital replantation in the United States [1,8-10,16,17].

We hypothesize that the declining success rates for digit replantation in the United States, while multifactorial, may be due to the lack of replantation experience of many US hand surgeons. We therefore believe that it is important to understand the role experience plays in digital replantation surgery. As in any technical task, microvascular surgery requires a very specific skill set that has to be learned over time. While there have been descriptions of surgical learning curves in many medical subspecialties such as cardiothoracic surgery and robot-assisted gynecological and general surgery [18-22], to date there are few studies describing the surgical learning curve for digit replant surgery in the United States.

Our hypothesis is that a learning curve exists in digital replant surgery and that a single surgeon would increase replant success over time due to increased experience [23]. The goals of this study were to outline the learning curve in replant surgery and seek to understand at what amount of time a surgeon becomes proficient in replant surgery.

This work was previously presented at the 2015 ASSH Annual Meeting in Seattle, WA.

## Materials and methods

Patient selection

Data was retrospectively reviewed from a prospectively collected trauma database from a single surgeon over a 10-year period. Study collection started on the first day of hand surgery practice following an accredited hand fellowship until 10 years into clinical practice. All digital replantation procedures during this time were included in the study. Approval from our institution’s human investigative committee was obtained.

Study variables

The primary outcome measure of the study was the success or failure of the digital replantation procedure. Patient variables measured in the study included the age of the patient, date of surgery (to determine the years since fellowship), finger or thumb replantation, zone of injury, and mechanism of injury (i.e., clean cut, crush, or avulsion). It was also hypothesized that the success or failure of digital replantation would depend on the overall microsurgery experience of the surgeon obtained from all microvascular cases. In addition to the above variables, the total hours underneath the microscope for all procedures (including replants, but primarily from microvascular work for free flap coverage and hand revascularization procedures) would be correlated with each replantation. This would give not only a time point from the fellowship as a marker of the learning curve but also an actual measurable variable of overall surgical experience. The total time under the scope was calculated by assigning an average operative time to particular procedures associated with each particular microvascular current procedural terminology (CPT) code. This estimation was for operative time underneath the scope and not total surgical time. All microsurgery-related CPT codes for the first 10 years in the surgeon’s practice were then retrospectively analyzed and total hours under the scope at the time of occurrence of each replantation was calculated.

Statistical analysis

Fisher’s exact test was used to determine the difference in percentage of replant survival across years. A linear regression model of survival probability was used to show increased survival over time. A cutoff point was determined by using sequential odds ratios (ORs) to find an inflection point in survival outcome. Finally, Pearson correlation, chi-square test, and ORs were used to determine relationships between study variables as appropriate. This study was approved as a retrospective review by our institutional review board at Banner Health #00002630.

## Results

Forty-six digital replants were performed over the 10-year study period. The average age at replantation was 33.9 ± 15.1 years, with an age range of 13 to 65 years. Twenty-four (52%) fingers and 22 (48%) thumbs were replanted. A majority of injuries occurred in zones one and two of the distal digits, with 23 (50%) injuries in zone one, 21 (46%) in zone two, and one (4%) in zone three. Injuries most often occurred due to saw/sharp cuts accounting for 31 (67%) of injuries, with 11 (24%) rope/avulsion injuries, and four (9%) crush injuries. Overall, 38 (83%) digit replants were successful and eight (17%) were unsuccessful (Table 1).

**Table 1:** Patient characteristics of the study population. The location was if the amputation injury was in the finger or thumb. Zone of injury refers to 1 (distal to the flexor digitorum profundus insertion) and 2 (proximal to the flexor digitorum profundus insertion).

Patient Characteristics	Success	Failure	Survival Rate
Age (years, average)	28	35	
Location			
Finger	22	2	91.60%
Thumb	16	6	72.70%
Zone of Injury			
I	20	3	86.90%
II	16	5	76.20
Unknown	1	0	100%
Mechanism			
Saw/Sharp	26	5	83.80%
Rope/Avulsion	9	2	81.80%
Crush/Trauma	3	1	75%

Observing the learning curve

**Figure 1:**
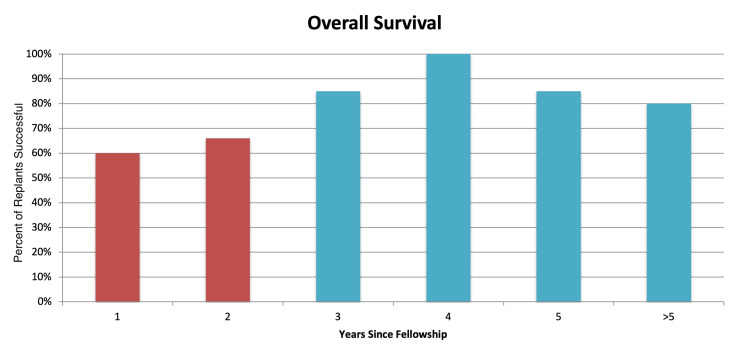
Overall replant survival by years in practice. Red represents success rates before 100 hours of microvascular experience, while blue is after 100 hours of experience. Chi-square test; p < 0.05

**Figure 2:**
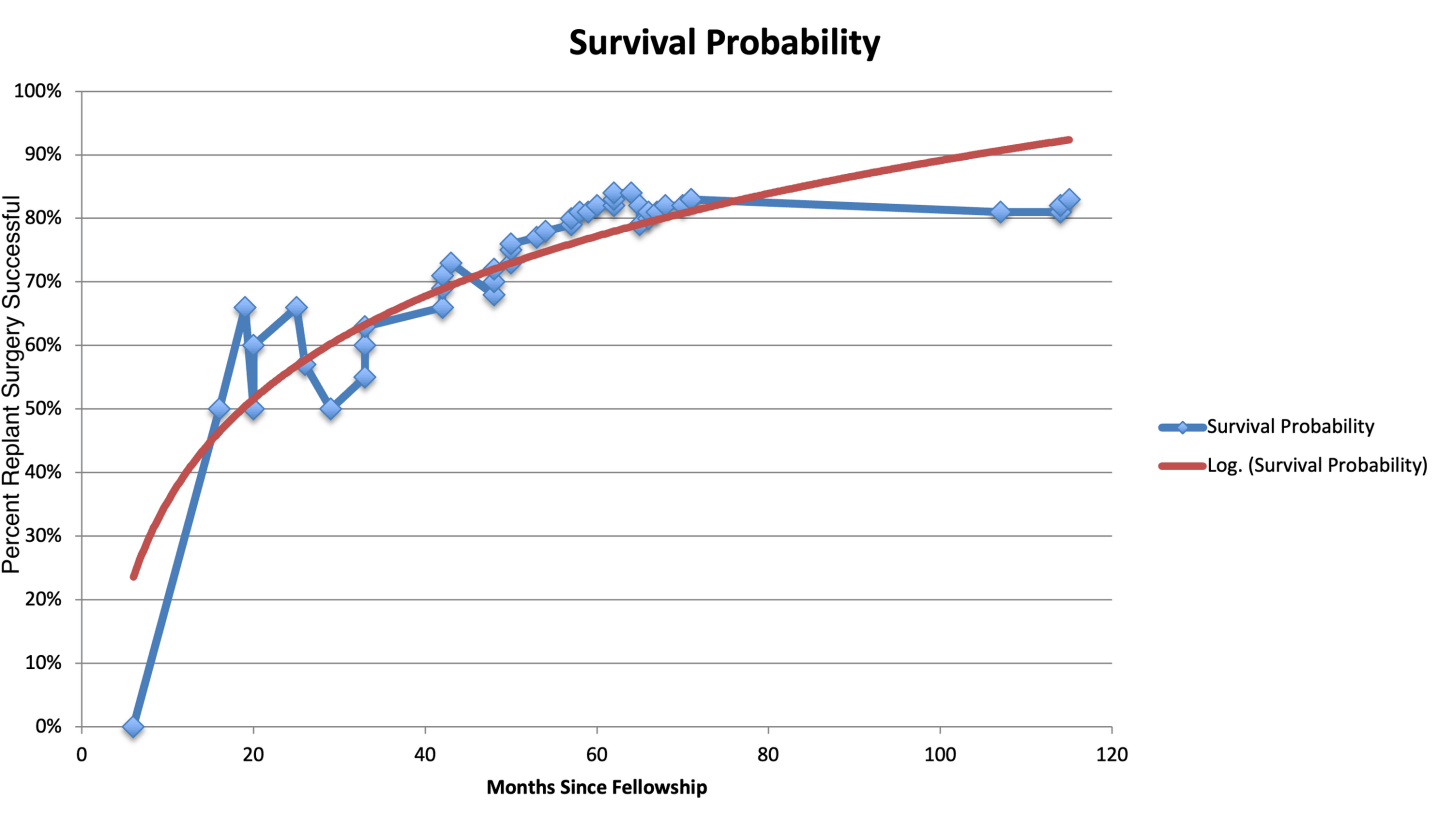
Point probability of replant survival by hours of microvascular experience. The blue line represents actual values from the study, red line represents the log fit estimate line. A log fit line is offered to better estimate the population level effect across surgeons. Linear regression model of survival probability; p < 0.05.

In this study, the surgeon performed 916 hours under the microscope from 1,565 microvascular procedures in a 10-year period. Hours under the scope significantly increased (R2=0.98, p<0.001) as months since fellowship increased showing a constant increase in overall scope usage over the 10-year period. There was a statistically significant increase in the overall survival rate as years since fellowship increased (Fisher’s exact test, p=0.04). In the surgeon’s first few years of practice, the overall success rate climbed incrementally: 60%, 66%, 85%, and 100% (Figure 1). The overall success rate for the entire study was 83%. In addition, a linear regression model of survival probability showed a statistically significant increase in survival probability over time, showing improved outcomes with increased surgical experience (p<0.001; Figure 2).

Time to surgical proficiency

**Figure 3:**
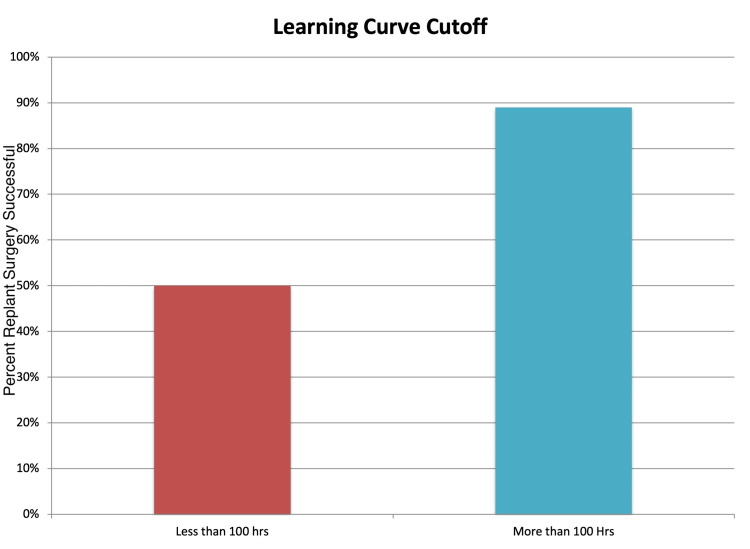
Digital replant survival rates before and after 100 hours of microvascular experience. Red represents before 100 hours of experience and blue represents after. Chi-square; p < 0.05.

Sequential ORs found an inflection point in the odds of replant survival around 100 hours of microvascular experience. Prior to 100 hours under the scope the surgeon had a survival rate of 50%. After 100 hours under the scope, the survival rate significantly increased to 89% (chi-square, p=0.02) (Figure 3). The OR of survival showed that after the surgeon had completed 100 hours under the scope, replant success was 8.5 times more likely to result in success (OR 8.5, 95% CI 1.5-47.9). One hundred operating hours under the scope correlated to 2.4 years in clinical practice since fellowship.

Correlating patient variables and outcomes

Patient variables were correlated with outcomes to determine the role of age, finger vs thumb, zone of injury, and mechanism of injury (Table 2). There was no difference in injury outcome based on the age of the patient, location of injury, or type of injury. However, patients with finger injuries showed a non-significant four times increase in odds of success of having a successful replant than those with thumb injuries (OR 4.12, 95% CI 0.7-23.2). Zone I amputations showed a non-significant two-fold increase in odds of survival over zone II/III amputations (OR 1.96, 95% CI 0.4-9.1). Finally, patients with clean-cut injuries showed a non-significant increase of two-fold in odds of survival than crush or avulsion injuries (OR 1.3, 95% CI 0.3-6.4).

**Table 2:** Odds ratios of replant survival for variables in the study. Odds ratios represent the odds of success with finger versus thumb, odds of success with zone 1 (distal to flexor digitorum profundus insertion) versus zone 2 (proximal), odds of success after 100 hours of experience, and odds of success with saw/sharp injury mechanism versus crush/avulsion.

Variable	Odds Ratio	95% Confidence Interval
Finger vs thumb	4.5	0.73-23.2
Zone 1 vs zone 2	0.51	0.11-2.5
100-hour cutoff	8.5	1.5-47.9
Saw/sharp vs crush/avulsion	0.77	0.16-3.8

## Discussion

In this study, the outcomes of 46 digital replants from one surgeon were followed from completion of fellowship to 10 years in practice. The surgeon showed significant improvement over the course of the study. A significantly improved clinical proficiency was reached after 100 hours of microvascular experience, which occurred after 2.4 years in practice. After an initial steep learning curve, the surgeon had significantly increased outcomes, with an 8.5 times greater likelihood of replant success.

The finding that experience is key to digital implant success will likely not come as a surprise. The outcome of a microvascular anastomosis is binary; it will either be a complete success or a complete failure. While a majority of hand surgeons may not frequently work under the operating microscope, virtually all are formally trained to replant amputated digits. Yet, recent investigations have shown increasing failure rates in digital replantation surgery [4,5,10,11,24]. The reason for this trend is unclear, but factors related to the patient or injury itself, such as mechanism of trauma, ischemia time, or zone of injury, do not appear as a likely explanation [4,5,11]. The present study was designed to investigate one specific risk factor related to the surgeon, by correlating microsurgical experience and failure rates. These findings suggest that it is possible that one of the most important factors in digital replant success is the level of experience of the surgeon and the volume of similar cases managed by the surgeon.

Surgical experience with digital replantation in the United States is a hot topic issue, especially in light of new pay-for-performance concepts and cost pressures in healthcare. In a 2007 survey among its members, the American Society for Surgery of the Hand (ASSH) found that 80% of respondents reported five or fewer replantation attempts yearly [16]. We are unaware of the members’ total microvascular surgery volume, but the report does not suggest an actual shortage of surgeons willing to perform microvascular procedures [9,10,15]. A more valid explanatory model for a national decline in success rates may be that not enough patients are referred to centers where microvascular reconstructive surgery is performed on a regular basis [9,16,17,25,26].

Many factors define the dispersion of potential candidates for digital replantation in the USA, and thus also the odds for satisfactory outcomes. Referral patterns that were traditionally driven by the availability and ability of surgeons are also increasingly influenced by large hospital systems discouraging referrals out of network. A problem with this model is that not all networks can offer continuing microvascular expertise for hand trauma since a hand surgeon’s practice is subject to changes over time (e.g., it is not uncommon that younger surgeons cover the emergency rooms and accept referrals for revascularization or replantation, only to cease taking such call after establishing their ideal outpatient practice). In this scenario, many patients are at risk of facing poor odds as a combined result of the presumed shape of a surgeon’s learning curve and the brief number of years that many hand surgeons have participated in microvascular calls [16].

The cost of digital replantation surgery can be calculated in a variety of ways, but a failed procedure is always a burden to the patient, the surgeon, and the insurer. In that perspective, some authors have recommended the development of centers of excellence to address issues of national referral patterns and increase referrals to high-volume hospitals and surgeons with increased replant success rates. Yet, the current study suggests that one of the possible factors affecting replant success is the level of experience of the surgeon. It may be that in the hands of an experienced surgeon digital replantation surgery can be safely and successfully performed in any facility that provides an operating microscope and a set of relatively simple instruments. For that reason, what could conceivably be depicted as an already existing national center of excellence for digital replantation in the USA is not a brick-and-mortar building, but a network of surgeons who are willing to commit to referral patterns, documentation, and reporting of results. We believe that there is good reason to better define this network, and we offer the present case study as an introduction to further discussion.

This study has some limitations. While the reported 46 consecutive cases are a reasonable volume for one surgeon, the numbers are relatively low for comparative statistical calculations such as ORs and correlation coefficients and therefore have resulted in large confidence intervals in this study when comparing patient variables and outcomes. However, this is offset by the benefits of comparing outcomes from a large cohort of replants from a single surgeon thereby minimizing bias from multiple surgical techniques and centers. A bias in patient selection is very evident given that there were as many thumbs replanted as all other fingers. This is a known bias. This arises as choosing to replant a finger is a much more complicated clinical decision that involves not only a discussion on indications, but also patient desire, patient occupation, and factors such as the patient’s ability to cooperate and endure the complicated postoperative replantation course. On the other hand, almost all thumbs are given at least a shot at a replant procedure due to the biomechanical necessity to hand function.

## Conclusions

Overall, declining success rates of replantation in the United States need a multi-modal approach to resolution, and we believe one major stepping stone could be the formation of centers of excellence for digital replantation. These centers of excellence would not be bound by brick-and-mortar buildings, but defined by a group of surgeons (institutional or private) who want to perform replants and agree on referral patterns. To achieve this goal, the cooperation of large hospital systems and contracting groups will be required. However, if we are able to do so on a regional basis, centers of excellence could allow surgeons who want to perform replants to develop and maintain clinical mastery and provide proper training to overcome the steep learning curve in digital replantation.
